# Upscaling of Apple By-Product by Utilising Apple Seed Protein as a Novel Wall Material for Encapsulation of Chlorogenic Acid as Model Bioactive Compound

**DOI:** 10.3390/foods11223702

**Published:** 2022-11-18

**Authors:** Asir Gani, Zanoor Ul Ashraf, Asima Shah, Azza Silotry Naik, Idrees Ahmed Wani, Adil Gani

**Affiliations:** 1 Department of Food Science and Technology, University of Kashmir, Srinagar 190006, India; 2 Food Science and Environmental Health, Technological University Dublin, D07 ADY7 Dublin, Ireland

**Keywords:** nanoencapsulation, protein, chlorogenic acid, release behaviour, antidiabetic, antioxidant

## Abstract

Encapsulation is a versatile technique used to protect sensitive bioactive compounds under gastrointestinal conditions. In this study, nanoencapsulation of chlorogenic acid into the apple seed protein matrix was performed using the green technique ultrasonication to protect it from harsh gastric conditions and increase its biological activity and bioavailability upon digestion. Both nano (Nano-Chl) and native capsules (NT-Chl) were characterised by particle size, charge, structure, and morphology. The encapsulation efficiency, release behaviour, antioxidant and antidiabetic properties were also evaluated. The experimental results show that the particle size of the NT-Chl and Nano-Chl was found in the range of 1.4 µm to 708 nm. The encapsulation efficiency was found to be 69% and 80% for NT-Chl and Nano-Chl, respectively. Furthermore, an in vitro digestion study revealed that Nano-Chl showed controlled-release behaviour under simulated intestinal conditions in comparison to NT-Chl. Moreover, Nano-Chl showed enhanced antioxidant and antidiabetic activity in comparison to NT-Chl after simulated digestion. It was concluded that the protein from apple seeds could be utilised as a functional ingredient itself or as a wall material for the encapsulation of sensitive bioactive compounds. Furthermore, these encapsulated particles can be fortified into different food formulations for the development of functional food.

## 1. Introduction

Chlorogenic acid is a polyphenolic compound existing widely in spices, fruits, vegetables, green coffee, etc., with green coffee being the richest source. [1]. It exhibits various biological properties such as anticancer, antilipidemic, anti-inflammatory, antioxidant, antidiabetic, and neuroprotective properties [2,3]. As a result, there is a growing consciousness of the utilisation of chlorogenic acid as a functional food ingredient in different formulations. Despite its proven pharmaceutical properties, the use of chlorogenic acid is limited in food systems because of low bioavailability and stability in the food. Moreover, it undergoes deleterious reactions under processing and storage conditions [4]. Furthermore, it is hydrolysed to caffeic acid and quinic acid in the intestine; hence, it is unstable [5]. Thus, encapsulation is an effective approach to overcome these obstacles and ensure its retention without influencing the quality of the food carrier. Encapsulation involves coating the active ingredients within wall material to protect it from the harsh gastric conditions. Furthermore, previous studies reported the encapsulation of chlorogenic acid into chitosan and cyclodextrin. However, no study has been reported on the encapsulation of chlorogenic acid into protein-based wall material [6,7]. Therefore, this study is the first report on the encapsulation of chlorogenic acid using apple protein as a delivery system. Different types of protein, such as whey proteins, soy proteins, vegetable proteins, and zein proteins, have been utilised as a sustainable delivery vehicle for the different sensitive bioactive ingredients [8,9,10]. Protein-based wall material is regarded as a safe, ecofriendly, and flexible carrier to entrap hydrophilic and hydrophobic bioactive ingredients [11,12]. Moreover, apple seed is a by-product of the apple juice industry, generally regarded as waste [13]. Apple seeds consist of a high quantity of proteins (49.5 g/100 g), lipids (24 g/100 g), dietary fibre, and other phenols that make them a potential food source for consumption [14]. In order to meet the spirit of sustainable development goals and zero carbon footprints, it is desirable to exploit apple seed as a sustainable protein source for use in food systems. Therefore, it is gaining importance in protein-based delivery systems as a wall material due to its excellent physiochemical and nutraceutical properties [13].

The present study was planned to nanoencapsulate chlorogenic acid into the apple protein as a wall material and analyse its nutraceutical potential, release behaviour, and encapsulation efficiency. Furthermore, this study opens ways to utilise apple seed protein itself as a functional ingredient or as a wall material for encapsulating bioactive ingredients. Moreover, developed native- and nano-apple protein-loaded chlorogenic acids can have incredible food and pharmaceutical uses as protein supplements.

## 2. Materials and Methods

### 2.1. Materials

Apple seeds were procured from FIL Industries, Pvt. Ltd., Srinagar, Jammu and Kashmir, India. The samples were cleaned, dried, dehulled, and then stored at 4 °C in airtight containers. The apple flour obtained was coarsely ground using a domestic blender. The chemicals and enzymes, such as α-amylase, α-glucosidase, pancreatin, pepsin, bile salts, DPPH, etc., used in this study were purchased from Sigma-Aldrich (Saint Louis, MO, USA).

### 2.2. Extraction of Protein

Briefly, defatted apple seed flour was dissolved in deionised water (pH 9–10.5) and stirred for 2 h at room temperature (25 °C) for the complete solubilisation of proteins [13]. This was followed by centrifugation at 1600× *g* for 10 min. The supernatant obtained was precipitated at the isoelectric point (pH 4.5) using 1 N HCl and re-centrifuged to recover the protein (pellet). The protein was freeze-dried and stored at −4 °C till use. The protein content in the freeze-dried sample was estimated to be 95.4 ± 0.54% on a dry-weight-basis (AOAC 1997) [13].

### 2.3. Preparation of Nanoparticles

Apple seed protein nanoparticles were prepared following the method of Shah et al. [15]. Briefly, apple seed protein dissolved in phosphate buffer solution (pH 7) was ultrasonicated at a frequency of 40 KHz for 25 min with a pause of 5 min. An ice bath was used throughout the sonication process so that the temperature remained constant (20–30 °C). The final mixture was then freeze-dried to obtain powder and stored at −20 °C for further use.

### 2.4. Encapsulation

The encapsulation of chlorogenic acid into protein nanoparticles was performed following the procedure of Shah et al. [13]. Both native (N-Protein) and nanoparticles (Us-Protein) of apple seed protein were dissolved in phosphate buffer solution (pH 7.2) and stirred continuously until a 10% solution was formed. This was followed by drop-wise addition of chlorogenic acid (5 mg/mL) dissolved in ethanol into apple seed protein solution. The chlorogenic acid loaded in Us-Protein was then ultrasonicated at 30 kHz for 10 min with a pause of 5 min at the temperature of 25 °C. After ultrasonication, both native- and nanoencapsulated solutions were freeze-dried and labelled as NT-Chl and Nano-Chl for chlorogenic acid encapsulated into native- and nano-apple-protein.

### 2.5. Analysis

#### 2.5.1. Swelling Index

The swelling index (SI) of the encapsulated samples was conducted following the method of Shah et al. [13]. Encapsulated capsules weighing 50 mg were dissolved in 20 mL of phosphate buffer saline at varying pH values (3, 4, and 7.5). The sample solution was incubated at 37 °C for two hours and centrifuged at 1000× *g* for 10 min. The supernatant was discarded, and the swollen mass (pellet) was removed, slightly dried, and reweighed. The swelling index (SI) was calculated as follows:

SI (%) = M_s_ − M_d_/M_d_ × 100

where M_s_ = the weight of the swollen sample and M_d_ = the weight of the dried sample.

#### 2.5.2. Particle Characterisation

The particle characteristics such as particle size (PI), polydispersity index (PDI), and zeta potential (ZP) were evaluated using dynamic light scattering (DLS) (Anton Paar, Litesizer). Both native- (NT-Chl) and nanoencapsulated (Nano-Chl) capsules were dispersed in distilled water (0.02%), followed by sonication for 10 min at 20 kHz in a sonicator bath. The sonicated solution was then analysed for particle size (PI), polydispersity index (PDI), and zeta potential (ZP).

#### 2.5.3. Microstructural Analysis

The microstructural analysis of the samples (NT-Chl and Nano-Chl) was visualised using a scanning electron microscope (Hitachi S-300H-Tokyo, Japan) at a voltage of 15 kV. The sample was placed on the aluminium stubs coated with gold and investigated for morphological characteristics under vacuum conditions.

#### 2.5.4. Fourier Transform Infrared Spectroscopy (FTIR)

The structural changes in the NT-Chl and Nano-Chl capsules were evaluated using FTIR (CARY 630, Agilent Technologies, Santa Clara, CA, USA). The FTIR spectra were collected at wavelengths ranging from 4000–400 cm^−1^ at 2 cm^−1^ of resolution.

#### 2.5.5. Encapsulation Efficiency (EE)

The encapsulation efficiency (EE) of the capsules was performed following the method of Gani et al. [15]. Briefly, 10 mg of NT-Chl and Nano-Chl was washed with 5 mL of distilled water and centrifuged for 10 min to remove the chlorogenic acid adhered to the protein surface. The supernatant was thrown away, and the pellet was dissolved in 20 mL of deionised water and incubated for 24 h with continuous stirring at 37 °C. After incubation, the whole solution was centrifuged at 1000× *g* for 10 min, and the absorbance of the supernatant was taken at 327 nm. EE was calculated by using Equation (1) y = 0.029x + 0.014 R^2^ = 0.904:

$$\mathrm{EE}\;\left(\%\right)=\frac{\mathrm{Amount}\;\mathrm{of}\;\mathrm{chlorogenic}\;\mathrm{acid}\;\mathrm{encapsulated}}{\mathrm{Amount}\;\mathrm{of}\;\mathrm{chlorogenic}\;\mathrm{acid}\;\mathrm{added}}\times 10$$

### 2.6. In Vitro Release Behaviour

The in vitro release study of encapsulated capsules was studied under simulated gastrointestinal conditions following the method of Jhan et al. [16]. NT-Chl and Nano-Chl (100 mg) capsules were dissolved in α-amylase solution (0.2%, pH 7.2), maintained at 37 °C and vortexed to simulate the mouth environment. The mixture was centrifuged at 5000× *g* for 5 min, and the release of chlorogenic acid was observed at 327 nm. The pellet was resuspended in 10 mL of simulated gastric juice containing pepsin (3 g/L) prepared in sterile NaCl solution (6g/L) and after constant stirring, aliquot (1 mL) was collected after 30 and 60 min of incubation. The mixture was centrifuged at 1500× *g* for 5 min. The supernatant was collected and release of cholorognic acid was observed under simulated gastric conditions. The recovered pellet from gastric phase was subjected to simulated intestinal conditions using pancreatin (10 g/L) and bile salts (3 g/L) prepared in phosphate buffer solution (pH 7.5) and incubated at 37 °C with gentle stirring. The whole reaction mixture was centrifuged at 5000× *g* for 5 min at the interval of 30, 60, and 120 min to evaluate the release of chlorogenic acid from the protein matrix under intestinal conditions. The amount of chlorogenic acid (%) released was measured and calculated as described in Section 2.5.4.

#### 2.6.1. Retention of Bioactivity

The encapsulated capsules (NT-Chl and Nano-Chl) were evaluated for bioactivity, such as antidiabetic and antioxidant properties, to study the effect of the encapsulation process on the retention of chlorogenic acid into apple seed protein matrix after in vitro simulated gastrointestinal digestion. Briefly, 100 mg of NT-Chl and Nano-Chl were dissolved in simulated gastric conditions (pepsin) and incubated at 37 °C for 1 h. This was followed by exposure to simulated intestinal conditions (pancreatic + bile salts) and re-incubated at 37 °C for 2 h. The mixture was centrifuged at 4000× *g* for 10 min and supernatant was stored at 4 °C for antioxidant and antidiabetic analysis.

##### In Vitro Antioxidant Assay

*DPPH Activity*

Free radical scavenging activity of the samples was performed following the method of Ashraf et al. [17]. Briefly, the reaction mixture containing DPPH solution was prepared in methanol (100 μL), sample (100 μL), and 800 μL of methanol to make the volume of 1 mL. The whole mixture was incubated in the dark for 40 min at a temperature of 20 °C, and absorbance of the sample was observed at 517 nm.

Percent inhibition was calculated using Equation (1):

(1) $$\mathrm{Inhibition}\;\left(\%\right)=\frac{S-b}{S}\times 100$$

where (S) is the control and (b) is the sample.

*Metal Chelating*

The metal-chelating activity of the encapsulated capsules was evaluated following the procedure of Ashraf et al. [17]. The reaction mixture consisting of a 1 mL sample solution, 1 mL acetate buffer (2 mM), and 1 mL FeCl_2_ (2 mM) was incubated for 30 min at room temperature. After incubation 0.25 mL ferrozine (5 mM) was added and incubated for 30 min. The absorbance of the sample mixture was taken at 700 nm, and % inhibition was calculated using Equation (1).

##### Antidiabetic Acitivity

*α-Amylase Inhibitory Activity*

The α-amylase inhibitory activity was performed following the method of Ashraf et al. [18]. The reaction mixture consisting of 50 μL of the enzyme (α-amylase 40 U/mL), 50 μL of the sample solution, and 10 μL of the sodium phosphate buffer (0.1 M, pH 6.9) was incubated for 10 min at 37 °C. After incubation, the starch solution (40 μL) was added as substrate and again incubated for 15 min. To terminate the reaction, HCl (40 μL) was added to the reaction mixture. This was followed by the addition of 5 mM iodine reagent, and colour change was observed. The absorbance of the sample was observed at 620 nm. The percent inhibition of α-amylase was calculated by following Equation (2):

% Inhibition = (P – A_o_)/(Ap – A_o_) × 100 (2)

where P signifies the absorbance of the sample/standard, A_o_ signifies the absorbance of the negative control, and Ap signifies the absorbance of the positive control.

*α-Glucosidase Inhibitory Activity*

Briefly, the incubation of the reaction mixture consisting of sample (25 μL), 0.1 M sodium phosphate (pH 6.8, 75 μL) and 25 μL α-glucosidase solution for 5 min was performed at 37 °C. Then, 25 μL of PNPG (15 mM) was added to start the enzymatic reaction and again incubated for 40 min. An amount of 100 μL sodium carbonate (0.2 M) was added to terminate the reaction. The hydrolysis of substrate by α-glucosidase was calculated at 405 nm. Inhibition (%) was evaluated using Equation (2).

### 2.7. Statistical Analysis

All the experiments were carried out in triplicates. The data were presented as mean ± standard deviation. Students *t*-test at 95% significance level was used to assess the difference between the mean using commercial statistical software (IBMSPSS statistics 21). 

## 3. Results and Discussion

### 3.1. Particle Size (PI), Polydispersity Index (PDI), and Zeta Potential (ZP)

The encapsulated capsules were characterised by size, polydispersity index, and surface charge using dynamic light scattering. Particle size is an important characteristic for understanding the behaviour of the encapsulated capsules, such as their encapsulation efficiency, release kinetics, solubility, and functionality [19]. The particle size of the native- (N-Protein) and nano-protein (Us-Protein) was found in the range of 1.2 µm to 484 nm, which significantly (*p* < 0.05) increased upon the encapsulation process as presented in Table 1. The size of native-encapsulated chlorogenic acid (NT-Chl) and nano-encapsulated chlorogenic acid (Nano-Chl) was found to be 1.5 µm and 708 nm, respectively. The increase in the size of the particles upon encapsulation could be due to the incorporation of chlorogenic acid into the protein matrix or the interaction of chlorogenic acid with the protein matrix [20]. The polydispersity index (PI) defines the heterogencity of the sample depending upon the size. The PI affects the dissolution and reactivity of the biopolymer in the solution, and its value for N-Protein, Us-Protein, NT-Chl, and Nano-Chl varied in the range from 0.1–0.4, indicating that the particles are monodisperse in nature and have good uniformity and stability. The nano size and smaller polydispersity indices of the capsule upon ultrasonication might be due to the cavitation process [21].

Zeta potential (ZP) represents the surface charge of the particles and provides information about attractive and repulsive forces between the particles. It influences the kinetic release and biological fate of nanoparticles [22]. As can be seen in Table 1, the ZP value was found to be in the range of −11 to −19 for wall materials (N-Protein and Us-Protein) and −18 to −24 for encapsulated capsules (NT-Chl and Nano-Chl). The negative value of ZP demonstrates the negative charge of the surface of encapsulated capsules resulting in preventing the agglomeration of particles.

### 3.2. Swelling Index (SI)

**Figure 1:**
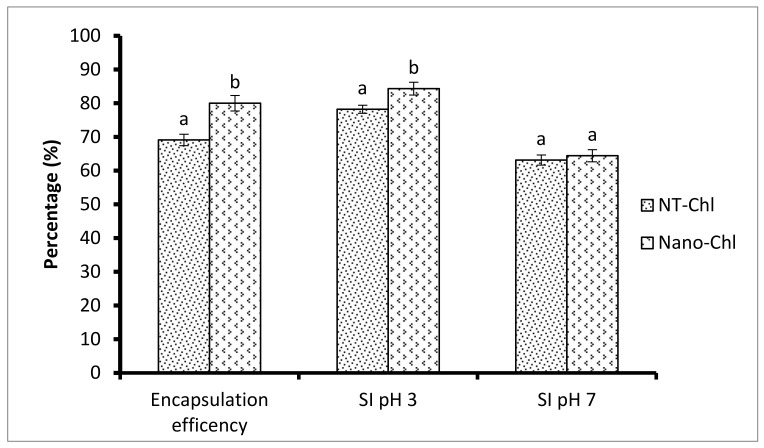
Encapsulation efficiency and swelling index at pH 3 and pH 7 of NT-Chl and Nano-Chl. Bars represent standard deviation (n = 3). Different letters on the bars representing different encapsulation method indicate significant differences (*p* < 0.05). Key: For caption, see Table 1.

Swelling behaviour is an important parameter that defines the ability of an encapsulant to entrap the sensitive ingredient under the simulated gastrointestinal tract conditions. The encapsulated capsules were rehydrated in a phosphate buffer solution at pH values 3 and 7.5 to mimic the gastrointestinal environment (Figure 1). It can be inferred that the higher the SI, the higher will be the resistance offered by the wall material to dissolution. Hence, the delayed release of chlorogenic acid will occur [23]. However, if the SI value of the encapsulated capsules is lower, the faster disintegration of the encapsulated capsules followed by the instant release of chlorogenic acid will occur [13]. At pH 3 and 4 (simulated gastric conditions), the encapsulated capsules (NT-Chl and Nano-Chl) exhibited an SI of 78.21% and 84.32%, respectively, indicating that the apple protein is stable and, therefore, higher the interruption in the release of chlorogenic acid due to higher SI. However, at pH 7.5 (simulated intestinal conditions), NT-Chl and Nano-Chl capsules exhibited a lower swelling index of 63.14% and 60.43%, respectively. This is attributed to the higher dissolution/disintegration of the apple protein in the alkaline medium followed by the release of chlorogenic acid [15]. These results are in accordance with a previous study reported by Gani et al. [19] in which churpi protein was used as wall material for the encapsulation of resveratrol. These results show that the release of chlorogenic acid from the apple protein matrix is pH-dependent and starts its release in the intestines.

### 3.3. Encapsulation Efficiency

Encapsulation efficiency (EE) is an essential feature that is defined as the fraction of bioactive compound entrapped inside the biopolymer matrix. The EE of NT-Chl and Nano-Chl was found to be 69.1% and 80.3%, respectively, as shown in Figure 1. It was found that Nano-Chl showed significantly higher encapsulation efficiency in comparison to NT-Chl (*p* < 0.05). The enhanced EE of the Nano-Chl could be ascribed to the nanosized particles used for encapsulation. Fine particle sizes have good EE because of their ability to produce a film around the bioactive ingredient; hence, there is higher retention of chlorogenic acid [18,19]. Ariyarathna et al. [24] reported the somehow similar results of chickpea protein loaded with folate.

### 3.4. Scanning Electron Microscopy

**Figure 2:**
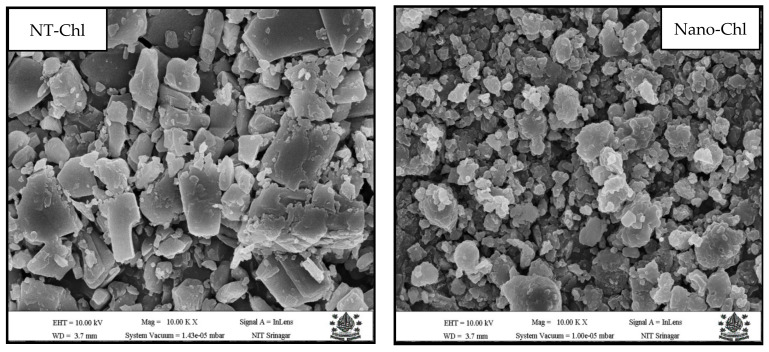
SEM images of NT-Chl and Nano-Chl. Key: For caption, see Table 1.

The morphological characteristics of NT-Chl and Nano-Chl were evaluated using SEM. NT-Chl showed a crystalline flake-like structure with greater dimensions, whereas Nano-Chl showed smaller, regular, and homogeneous structures. The ultrasonication process resulted in the disintegration of larger flake-like structures into finer regular structures (Figure 2) in the nanorange (Table 1). These morphological alterations have a significant impact on the bioactivity and release behaviour of bioactivities (Figures 4 and 5). Similar results were reported by Shao et al. [7] for chlorogenic acid encapsulated in beta-cyclo-dextrin. Moreover, Donsì et al. [25] reported similar results for Zein-based colloidal particles for encapsulation of epigallocatechin gallate.

### 3.5. FTIR

**Figure 3:**
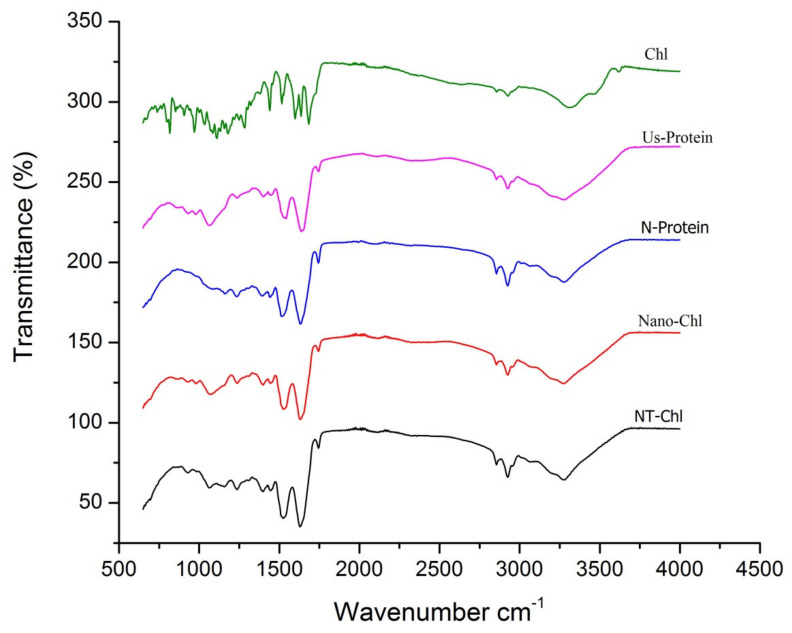
FTIR spectrum of Native apple seed protein (N-Protein), Ultrasonicated apple seed protein (Us-Protein), Chlorogenic acid (Chl), chlorogenic acid loaded in native apple seed protein (NT-Chl) and chlorogenic acid loaded in nano apple seed protein (Nano-Chl).

The FTIR analysis of N-Protein, US-Protein, NT-Chl, Nano-Chl, and chlorogenic acid is represented in Figure 3. The IR spectra of chlorogenic acid present the broad stretching peak at 3428 cm^−1^ representing OH vibrations. The peak at 1500–1750 cm^−1^ can be related to the C=C vibrations of an aromatic ring and 1250 cm^−1^ to the C-O broadening. The infrared spectra of apple seed protein (N-Protein and US-Protein) represent a wide band stretching of peaks from 3000–3400 cm^−1^ corresponding to the existence of amide A (N-H bonds), 1600–1750 cm^−1^ corresponding to amide I, 1400–1570 cm^−1^ corresponding to amide II, and 1050–1200 cm^−1^ corresponding to amide III regions, which is due to the C=O stretching and combination of C-N and N-H bending vibrations [13]. However, the encapsulated capsules (NT-Chl and Nano-Chl) showed a shift in intensity at the amide A and amide III zones in comparison to N-Protein and US-Protein. The presence of new peaks in the NT-Chl and Nano-Chl indicates the successful encapsulation of chlorogenic acid into apple seed protein. Likewise, these findings are in agreement with the study reported by Nallamuthu et al. [6] and confirm the interactions between the chlorogenic acid and amine groups of protein, signifying the efficacious entrapment of chlorogenic acid into the apple protein matrix. 

### 3.6. In Vitro Release Behaviour of Chlorogenic Acid

**Figure 4:**
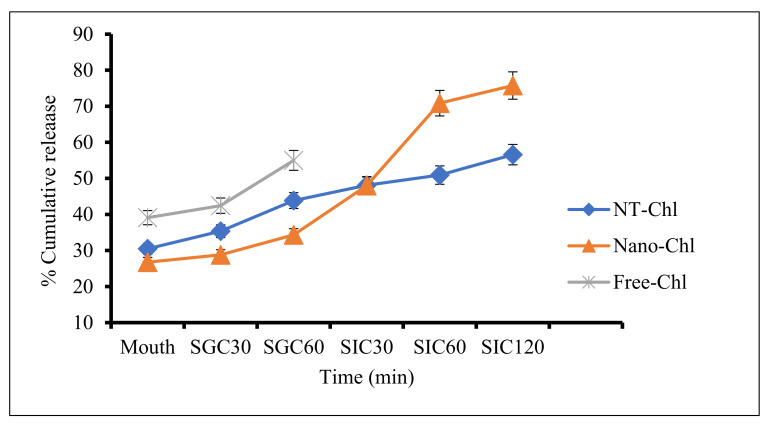
Release pattern of Free-Chl, NT-Chl, and Nano-Chl under simulated gastrointestinal conditions, where SGC (simulated gastric condition) is at 30 and 60 min of incubation and SIJ (simulated intestinal condition) is at 30, 60, and 120 min of incubation. Key: For caption, see Figure 3.

The purpose of the nanoencapsulation was to limit the degradation and abrupt release of chlorogenic acid in the simulated gastrointestinal conditions and retain its bioactivity at the targeted site (Figure 4). Chlorogenic acid in free form (Free-Chl), NT-Chl, and Nano-Chl capsules were exposed to in vitro simulated human digestion conditions, and their release pattern was investigated, as presented in Figure 4. In the oral phase, the quantity of chlorogenic acid released for Free-Chl, NT-Chl, and Nano-Chl was found to be 39.1%, 30.4%, and 26.7%, respectively. The initial release of chlorogenic acid from Free-Chl and nanoencapsulated capsules in the mouth can be attributed to chlorogenic acid adhered to proteins matrix with weak bonds [15,23]. In the simulated gastric conditions, the amount of chlorogenic acid released during the first 30 min from Free-Chl, NT-Chl, and Nano-Chl was found to be 42.44%, 35.5%, and 28.78%, respectively. However, after 60 min, the quantity of chlorogenic acid released for Free-Chl, NT-Chl, and Nano-Chl was found to be 55%, 43.8%, and 34.3%, respectively. The regulated release under gastric conditions can be attributed to the swelling of capsules and the prevention of disintegration of capsules. Free-Chl was found to be released fully under simulated gastric conditions [22]. Similar results were reported by Gani et al. [13]: Shah et al. [23]: Shah et al., [15] for the release of vitamin D_3_, rutin, and catechin from nanoencapsulated capsules. Moreover, Mohammadian et al. [26] described a similar release pattern with curcumin from mung bean protein isolate. Furthermore, under simulated intestinal conditions of 30, 60, and 120 min, the release of chlorogenic acid was found to be in the range of 48–56% for NT-Chl and 47–75 for Nano-Chl. These results reveal that the higher pH value under simulated intestinal conditions (alkaline medium) is responsible for the decreased swelling value of capsules, thereby resulting in the disintegration of capsules followed by an increased release of chlorogenic acid. The enhanced release from Nano-Chl may be due to the higher encapsulation efficiency of Nano-Chl (Figure 1). The results reveal good stability for chlorogenic-acid-loaded apple seed protein under harsh gastrointestinal conditions, thus making it an interesting vehicle for the delivery of bioactive molecules.

### 3.7. Antidiabetic Activity

**Figure 5:**
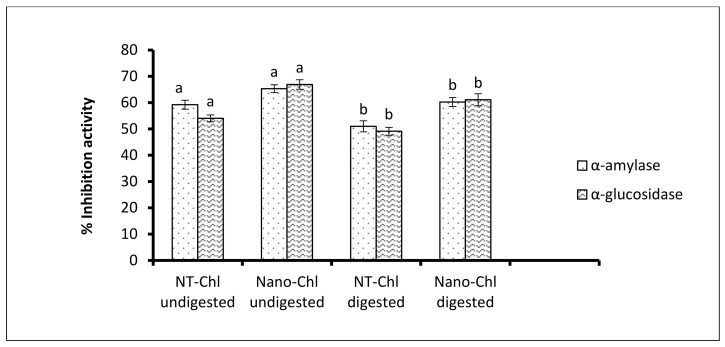
Antidiabetic activity of the NT-Chl and Nano-Chl. Bars represent standard deviation (n = 3). Different letters on the bars representing same enzyme treatments and encapsulation methods indicate significant differences (*p* < 0.05). Key: For caption, see Figure 3.

The antidiabetic activity of the nanoencapsulated capsules was evaluated using in vitro α-amylase and α-glucosidase inhibition activity. This involves the hydrolysis of starch molecules into sugar. Hence, inhibitors to this enzyme inhibit the digestion and absorption of dietary starches by the body, whereas α-glucosidase involves the hydrolysis of disaccharides to monosaccharides and the inhibition of α-glucosidase controls blood glucose and, thus, decreases postprandial plasma glucose levels in diabetics. The percent inhibition value of α- amylase and α-glucosidase varied significantly before and after digestion, as shown in Figure 5. Before digestion, the percent inhibition value of NT-Chl and Nano-Chl was found to be in the range of 59–65% for α-amylase and 54–67% for α-glucosidase. However, after digestion, it was found to be in the range of 50–60.9% for α-amylase and 49–61% for α-glucosidase. The results reveal that Nano-Chl showed higher antidiabetic activity in comparison to NT-Chl both before and after digestion, which is attributed to the particle size in the nanorange, which enhanced the inhibition activity of Nano-Chl. Furthermore, antidiabetic activity has been reportedly exhibited by chlorogenic acid and apple seed protein [13,14,27].

### 3.8. Antioxidant Activity

**Figure 6:**
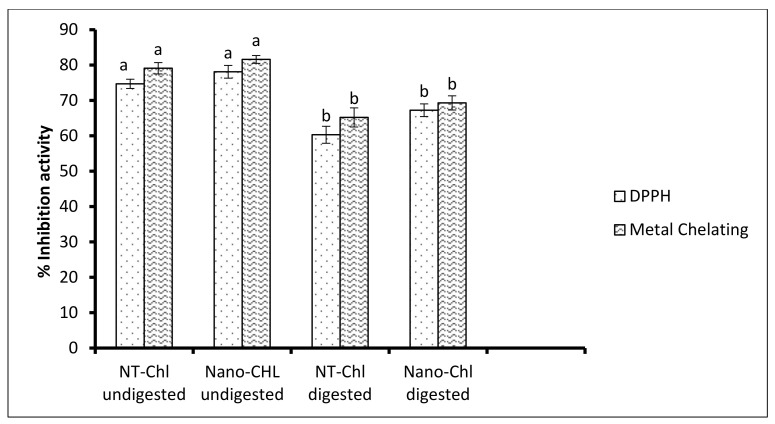
Antioxidant activity of the NT-Chl and Nano-Chl. Bars represent standard deviation (n = 3). Different letters on the bars representing same antioxidant assay and encapsulation methods indicate significant differences (*p* < 0.05). For caption, see Figure 3.

The antioxidant activity of NT-Chl and Nano-Chl was evaluated against free radical scavenging activity (DPPH) and metal-chelating activity before and after in vitro digestion, as shown in Figure 6. In free radical scavenging activity, the free radical accepts electrons to attain stability, corresponding to a change in colour. The inhibition activity (%) of NT-Chl and Nano-Chl varied significantly (*p* < 0.05) before and after digestion. Before digestion, it was found to be 64.7–78.1%; however, after digestion, it was found to be 60.3–67.23%. The hydroxyl groups and higher electron density on chlorogenic acid will easily provide electron for stabilizing free radical. However, in the case of metal-chelating activity, chelating agents inactivate metal ions and potentially inhibit the metal-dependent processes providing antioxidant activity. The percent inhibition activity of NT-Chl and Nano-Chl was evaluated before and after digestion. Before digestion, it was found to be in the range of 69.1–81.6%, and after digestion, it was found to be in the range of 65.2–69.3%. Overall, the results reveal that the nanoencapsulation process prevented the deterioration of chlorogenic acid, and a good amount of chlorogenic acid was retained at the targeted site. Nallamuthu et al. [6] similarly found the radical scavenging activity of chlorogenic acid loaded in chitosan nanoparticles [7].

## 4. Conclusions

This study involves the encapsulation process of chlorogenic acid, which showed enhanced bioavailability and bioactivity and provided a comparison between the micro- and nanoencapsulated systems of apple seed protein loaded with chlorogenic acid. Nano-Chl provided good EE in comparison to NT-Chl with sustainable releasing properties. The Nano-Chl also showed higher antioxidant and antidiabetic properties as compared to NT-Chl indicating that nano-delivery systems can be incorporated into different food and pharmaceutical uses for the increased delivery of chlorogenic acid. Moreover, this study provides an avenue for the utilisation of agro-waste as a novel source of protein for the encapsulation of sensitive bioactive compounds.

## Figures and Tables

**Table 1:** Particle size, polydispersity index and zeta potential of native protein, ultrasonicated protein and chlorogenic acid encapsulated in native protein and ultrasonicated protein.

	Native-Encapsulated		Nano-Encapsulated	
	N-Protein	NT-Chl	Us-Protein	Nano-Chl
Particle size	1.2 ± 0.2 µm ^b^	1.5 ± 0.1 µm ^a^	484 ± 1.5 nm ^b^	708 ±1.2 nm ^a^
Polydispersity index	0.1 ± 0.2 0.3 ^b^	0.3 ± 0.2 ^a^	0.23 ± 0.5 ^b^	0.4 ± 0.1 ^a^
Zeta potential (mV)	−11 ± 1.5 ^b^	−18 ± 0.2 ^a^	−19 ± 0.7 ^b^	−24 ± 1.7 ^a^

Results are mean ± SD. Means in the rows under same encapsulation method with different superscripts are statistically significant (*p* < 0.05). Native apple seed protein (N-Protein), Ultrasonicated apple seed protein (Us-Protein chlorogenic acid loaded in native apple seed protein (NT-Chl) and chlorogenic acid loaded in nano apple seed protein (Nano-Chl).

## Data Availability

Not applicable.
